# Spontaneous Pneumothorax as a Late Complication of Mild COVID-19 Infection: A Case Report

**DOI:** 10.7759/cureus.23294

**Published:** 2022-03-18

**Authors:** James Love, Rico Chenyek, Amanda Osta

**Affiliations:** 1 Department of Medicine, University of Illinois Chicago, Chicago, USA; 2 Department of Pediatrics, University of Illinois Chicago, Chicago, USA

**Keywords:** clinical complications of covid-19, mild covid-19, covid-19 vaccination, primary spontaneous pneumothorax, spontaneous pneumothorax, covid-19

## Abstract

Spontaneous pneumothorax (PTX) is a rare but life-threatening complication of lung injuries sustained from severe COVID-19 infection, most commonly associated with mechanical ventilation. Development of spontaneous PTX in patients after only mild COVID-19 infections not requiring hospitalization is even rarer. Here, we present the case of a 37-year-old male with spontaneous PTX secondary to a mild COVID-19 infection diagnosed one-month prior. A computed tomography (CT) scan of the chest revealed new air-filled cysts thought to be mediated by the inflammatory response to his acute infection, and his PTX was thought to be secondary to cyst wall rupture due to prolonged coughing. He was successfully treated with a chest tube and supplemental oxygen and, at a two-month follow-up, demonstrated clinical and radiographic improvement.

## Introduction

Since its outbreak in Wuhan, China, in late 2019, the novel 2019 coronavirus (COVID-19) has rapidly spread throughout the world and remains a significant burden to our healthcare systems despite the introduction of vaccines. While death rates have declined among the vaccinated, COVID-19 still affects thousands of Americans daily with a wide variety of symptoms and post-infectious complications ranging from mild flu-like symptoms to severe acute respiratory distress requiring mechanical ventilation [1]. 

Spontaneous pneumothorax (PTX) is a rare but life-threatening complication of lung injuries sustained from severe COVID-19 infection occurring in approximately 1% of hospitalized patients with COVID-19, though this is most commonly associated with mechanical ventilation due to barotrauma [2-5]. Development of spontaneous PTX in ambulatory patients without significant underlying lung disease after only mild COVID-19 infection not requiring hospitalization is even rarer, and its pathogenesis is poorly understood [6]. Here, we present such a case of spontaneous PTX secondary to a mild COVID-19 infection and highlight the importance of both prevention and early recognition followed by treatment of this life-threatening complication. 

## Case presentation

**Figure 1:**
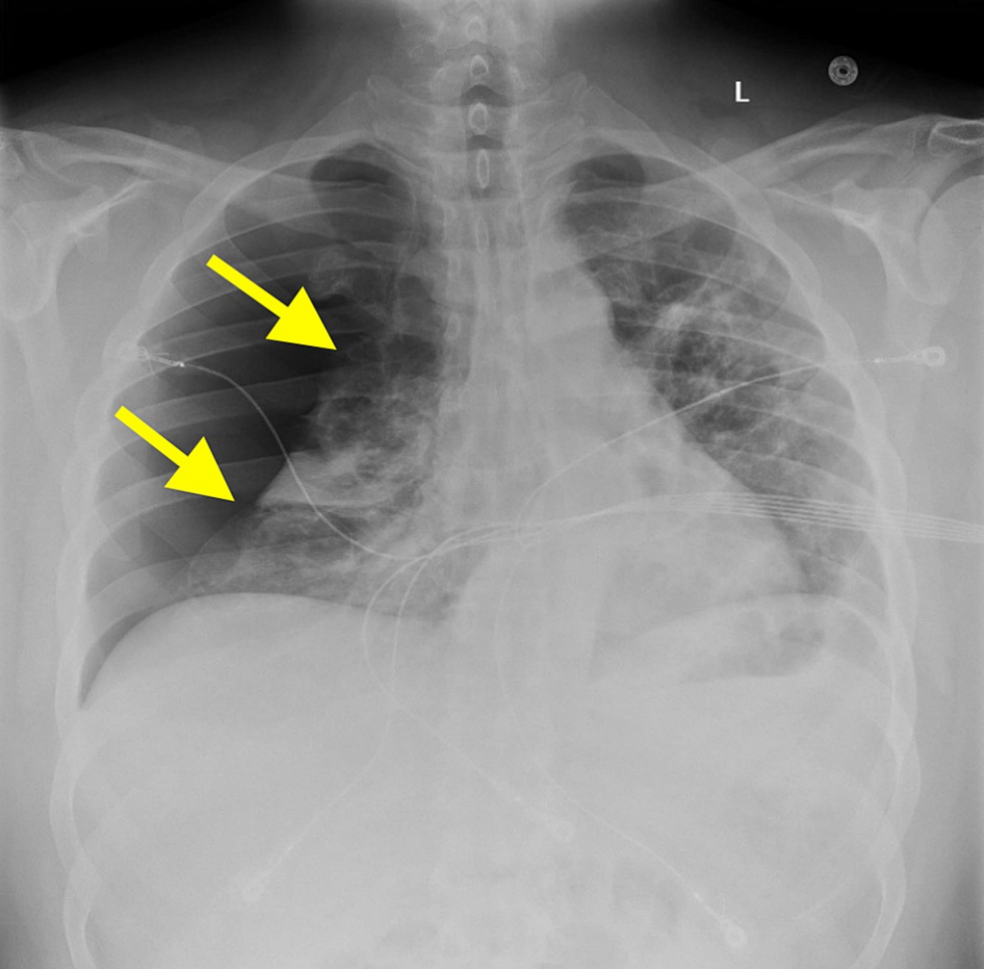
Chest x-ray on arrival demonstrating large right pneumothorax with right lung atelectasis.

A 37-year-old previously healthy male presented to the emergency department with acute-onset pleuritic chest pain, shortness of breath, and hemoptysis in the setting of a four-week history of cough and fatigue secondary to mild COVID-19 infection being treated outpatient with supportive measures in September 2021. His social history was negative for significant tobacco use or second-hand smoke exposure, and he and his partner had declined vaccination against COVID-19. On arrival, he was tachypneic and intermittently hypoxic to less than 88% and was placed on a 2L O2 nasal cannula. On exam, he had reduced right-sided breath sounds. Shown in Figure 1, chest X-ray (CXR) revealed a large right-sided PTX and subsequent placement of a pigtail chest tube with improvement in respiratory symptoms.

**Figure 2:**
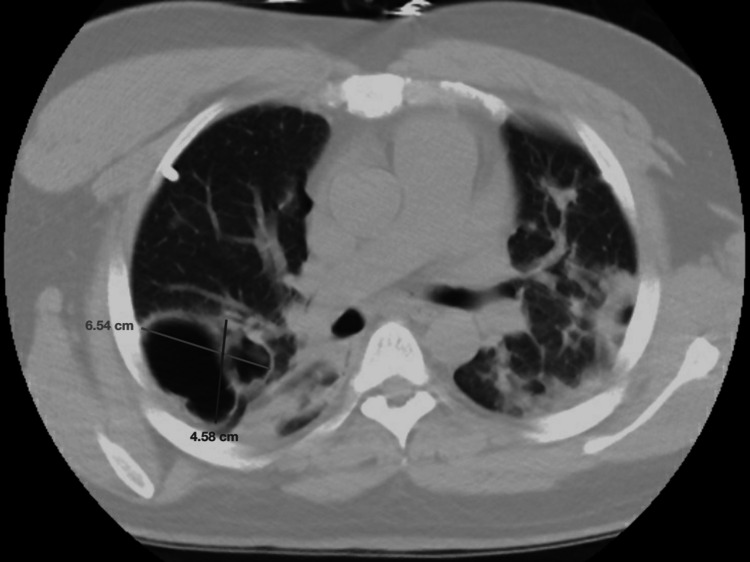
Computed tomography of the chest without contrast demonstrating loculated, air-filled, thick-walled cysts along the right major fissure measuring 6.5 x 4.6 x 3.0 cm.

Repeat CXR confirmed the resolution of the PTX, and he was admitted for further management. A computed tomography (CT) scan of his chest, shown in Figure 2, revealed large, loculated, thick-walled air-filled cysts along the right major fissure thought to be secondary to the inflammatory response to recent COVID-19 infection in the absence of prior imaging.

**Figure 3:**
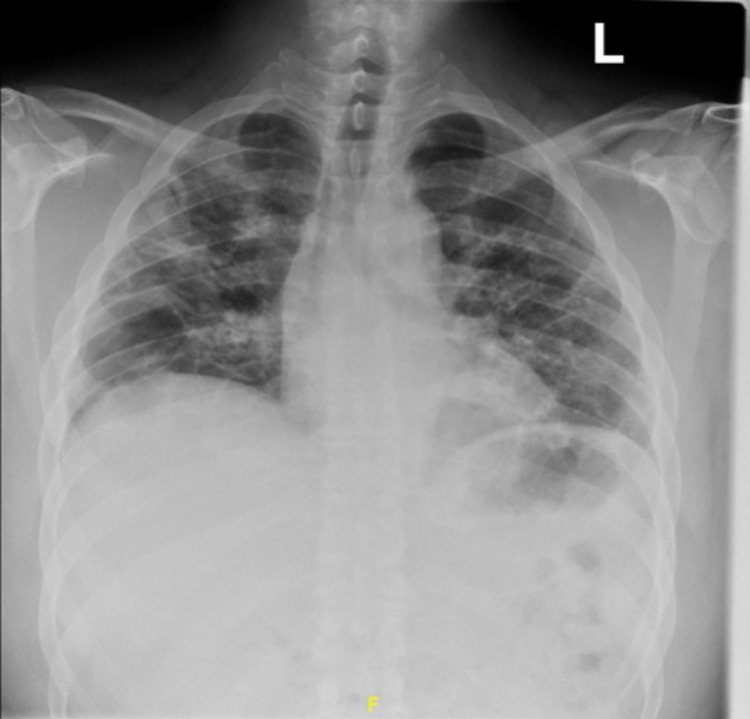
A chest X-ray was taken one month after hospitalization without clinical or radiographic signs of pneumothorax.

On day two of his admission, his chest tube was removed, supplemental oxygen was gradually weaned, and he consented to COVID-19 vaccination on the day of discharge after extensively shared decision-making with both the patient and his partner, who now not only encouraged him to get vaccinated but also expressed motivation to get vaccinated herself. At one-month follow-up, his coughing and pleuritic chest pain had improved, and his repeat CXR was stable without clinical or radiographic signs of PTX, shown in Figure 3.

**Figure 4:**
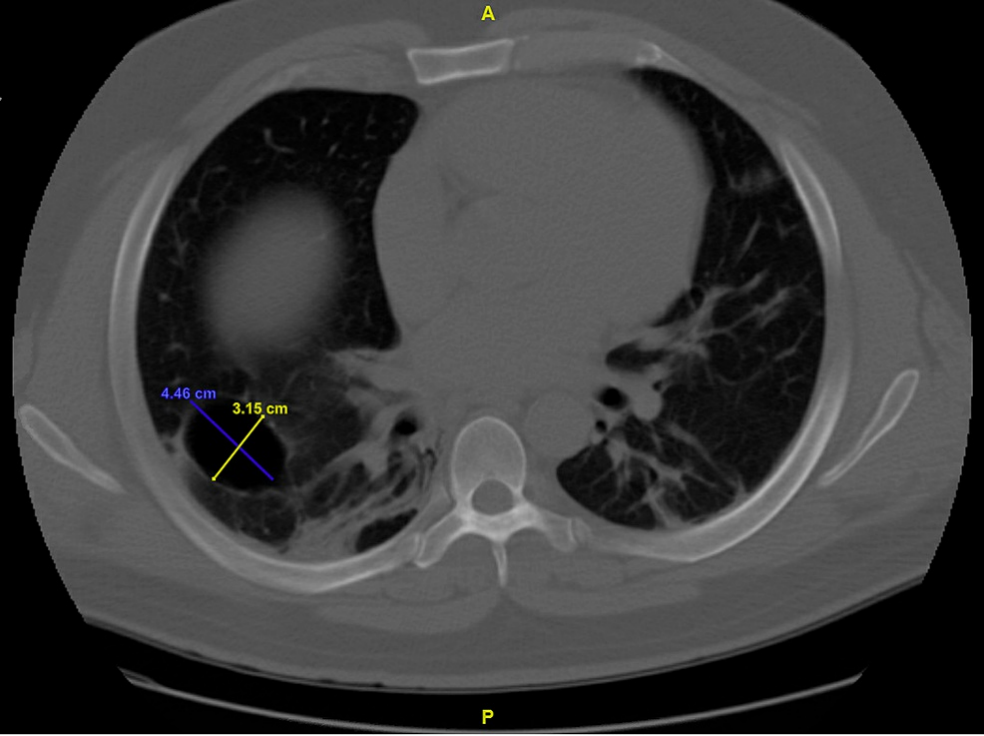
Computed tomography of the chest without contrast demonstrating improvement in overall size and wall thickness of cystic lung lesion along right major fissure at two-month follow-up, measuring 4.5 x 3.2 cm.

At two-month follow-up, his symptoms had nearly returned to his baseline, and repeat CT chest demonstrated improvement in his cystic parenchymal lesions, as shown in Figure 4.

## Discussion

We present a case of a spontaneous pneumothorax secondary to a mild case of COVID-19 pneumonia not requiring hospitalization in a previously healthy 37-year-old male. Although rare, this case illustrates not only the importance of vaccination but also the importance of physicians recognizing spontaneous PTX as a late complication of COVID-19 infection, even in patients with only mild symptoms who do not require inpatient admission.

Spontaneous pneumothorax is defined as the accumulation of air between the visceral and parietal pleura of the lungs, and its incidence in COVID-19 is extremely rare in the absence of barotrauma from mechanical ventilation [2]. Due to the novel nature of the virus and the rarity of PTX in patients with a history of mild COVID-19, the exact pathogenesis of spontaneous PTX is yet to be fully elucidated. Ultimately several proposed mechanisms may increase the likelihood of developing spontaneous PTX. Its pathogenesis most likely involves structural alterations in the lung parenchyma found in the lung tissues of patients with severe COVID-19 infection, which disrupt surface proteins and promote hypercoagulability via NF-kB/NFk-B2 pathway activation [7]. With downregulation of surfactant, loss of extracellular matrix and basement membrane, and promotion of hypercoagulability, this suggests COVID-19 promotes hypoxia and weakens lung parenchyma, thus predisposing a patient to develop a PTX.

Interestingly, in this case, our patient was found to have large cystic parenchymal changes on CT. Similar cystic lesions have been identified in patients with COVID-19, though these are typically found in the setting of severe infection, and it is unclear whether they are mediated by direct viral injury or whether this is secondary to factors such as prolonged mechanical ventilation, acute respiratory distress syndrome, or underlying lung disease [8-9]. While our patient did not have prior cross-sectional imaging, it is believed that his cystic parenchymal findings were new and secondary to his COVID-19 infection following comparison with other documented cases in which imaging before COVID-19 infection was normal [8]. Further strengthening this belief is the fact that our patient’s clinical improvement correlated with radiographic improvement in his cystic lesions at a two-month follow-up. In the presence of parenchymal inflammation and COVID-19-mediated alveolar damage, increased intrathoracic pressure from repetitive coughing may also have contributed to the development of spontaneous PTX, though typically patients documented to have developed PTX after repetitive coughing also had experienced more severe forms of COVID-19 [6,10]. More research is needed to understand the prevalence and significance of similar radiographic and clinical findings in patients with only mild infections, which was found in our patient.

Our patient was admitted for acute hypoxic respiratory failure secondary to spontaneous PTX in the setting of COVID-19 pneumonia. Cell-mediated and cytokine damage from his mild COVID-19 infection likely led to the formation of parenchymal cystic lesions, which ruptured due to repetitive coughing, leading to a spontaneous PTX. He was successfully treated with chest tube decompression supplemental oxygen and was vaccinated before discharge. This case highlights an extremely rare complication of a virus that has caused a global pandemic. Of note, it is unknown whether this complication has occurred in patients who are vaccinated against COVID-19. Nevertheless, as we learn more about late complications of COVID-19 infection, this case stresses the importance of widespread preventative measures to end the COVID-19 pandemic. It also speaks to the utility of discussing spontaneous PTX as a potential late sequela of mild COVID-19 infection in patient education concerning vaccination against COVID-19. 

## Conclusions

Although rare, this case illustrates the importance of physicians recognizing spontaneous PTX as a late complication of COVID-19 infection, even in patients with only mild symptoms who do not require inpatient admission. Additionally, while similar cystic lesions have been described and linked to COVID-19 infection, these are typically found in the setting of severe infection and have never been described in patients with mild COVID-19 infection until now. More research is needed to understand the prevalence and significance of similar radiographic findings in ambulatory patients with mild infection. 
